# QuantiFERON Gold-In-Tube for the diagnosis of mycobacterial tuberculosis infection in children under 5 years of age: A systematic review and meta-analysis

**DOI:** 10.1371/journal.pone.0295913

**Published:** 2024-01-02

**Authors:** Thomas Volkman, Visai Muruganandah, Hamish Graham, Shidan Tosif, Simon Stokes, Sarath Ranganathan

**Affiliations:** 1 Department of General Paediatrics (Refugee Health), Perth Children’s Hospital, Perth, Western Australia, Australia; 2 College of Medicine and Dentistry, James Cook University, Cairns, Queensland, Australia; 3 Children’s Emergency Department, The Prince Charles Hospital, Brisbane, Queensland, Australia; 4 Department of General Medicine, Royal Children’s Hospital Melbourne, Melbourne, Victoria, Australia; 5 Infection and Immunity, Murdoch Children’s Research Institute, Melbourne, Victoria, Australia; 6 Department of Paediatrics, The University of Melbourne, Melbourne, Victoria, Australia; 7 Department of General Paediatrics, Peninsula Health, Melbourne, Victoria, Australia; Shandong Public Health Clinical Center: Shandong Provincial Chest Hospital, CHINA

## Abstract

**Background:**

Previous meta-analysis regarding the performance of QuantiFERON Gold-In-Tube in children have yielded contrasting results. Emerging data in children younger than 5 years of age necessitates a new analysis.

**Methods:**

Systematic searches were conducted of MedLINE, EMBASE and Cochrane databases between 1998–2023. Pooled estimates of sensitivities and specificities of QFT-GIT compared to tuberculin skin test (TST) were calculated. The Kappa (k) coefficient was calculated for each study to determine the degree of congruence between TST and QFT-GIT results. Studies including patients co-infected with HIV or other immune compromising conditions or those treated with anti-tubercular treatment were excluded.

**Results:**

Seventeen studies (4335 patients) were included in quantitative analysis. All studies were conducted in middle to high income countries. They were conducted across 14 countries and 4 studies in countries with high TB incidence. The pooled sensitivity, specificity and DOR were 0.45 (0.42–0.48), 0.96 (0.96–0.97) and 18.84 (7.33–48.41) respectively. The ability of QFT-GIT to discriminate with disease and no disease was “good” as demonstrated by a summary receiver operating characteristic curve with area under curve of 0.7812. The average Kappa (k) co-efficient was 0.501 with a wide variety of values between studies (0.167 to 0.800).

**Conclusion:**

The findings of this meta-analysis support the judicious use of QFT-GIT in children 5 years and under, with caution as a sole test to exclude Tuberculosis in this age group. The heterogeneity and methodological quality of diagnostic studies limits the generalisability of results.

## Introduction

Tuberculosis remains a significant public health concern amongst the paediatric population. In 2020, children (aged <15 years) accounted for 11% of all new notified cases of TB worldwide [1]. Traditionally, infections with M. *tuberculosis* have been categorized into active and latent TB infection (LTBI), however more recently the World Health Organisation (WHO) has adopted the term “TB infection” to better indicate the continuum of the process following inhalation of bacilli that may lead to disease [2]. WHO modelling estimates that 7.5 million children (<15 years) are infected with *Mycobacterium tuberculosis* (M. tuberculosis) each year [2]. The risk of developing active TB after acute infection is high (19%) in children younger than 5 years [3]. Active disease, including pulmonary, meningitis and disseminated TB, especially in children under 3 years of age, is associated with high morbidity and mortality [4]. This can be greater in immunocompromised children, such as those living with HIV or malnutrition [5].

The ‘gold standard test’ for detection of TB infection in a mixed population of well and symptomatic children is challenging, especially in low incidence countries. Observing the cutaneous reaction to a mixture of mycobacterial peptides (PPD) by TST, was the mainstay of diagnosis of infection for over 100 years until 2002 when commercial Interferon-gamma release assay (IGRA) testing became available [6]. Limitations of the TST can be shortages in PPD, staff training requirements and the need for two attendances for insertion and recording of response. False-positive reaction following prior Bacillus Calmette-Guérin (BCG) vaccination, non-tuberculous mycobacterial species exposure, and false negatives in the setting of immunosuppression all occur [7]. Recent WHO guideline updates have included new antigen-based skin tests (TBSTs) as recommended tests due to potentially improved specificity [2].

IGRA have emerged as a commonly used tool for indicating tuberculosis infection since the advent of the second generation of the test- the QuantiFERON Gold In-Tube (QFT-GIT, Cellestis Ltd, Carnegie, Victoria, Australia). This includes a third antigen and only requires 3mls of blood compared to the 16mLs required for the original assay. Potential benefits include remaining unaffected by prior BCG administration, no boosting effect with repeat administration and a single blood draw. It has greatly improved the practicality of its use, and the additional antigen is thought to enhance its sensitivity without sacrificing specificity [8].

Understanding the limited evidence of true sensitivity of these tests and the caveats for their use (such as age) is difficult outside the specialist setting. Studies examining close TB contacts progression to TB disease according to their IGRA/TST status have concluded similar sensitivity but improved specificity of IGRA but have had very limited sample sizes in young children <6 years of age [9]. Cohort contact studies are also hampered by the duration of follow up time in discordant result cases to establish late disease diagnosis and therefore sensitivity.

Clinical diagnosis has traditionally comprised of a combination of determining clinical signs and symptoms, chest radiography, TST/IGRA and a positive contact history. Formulating a clinical diagnosis can be subjective and can vary between specialist clinicians. As such, a novel stand-alone test to diagnose childhood tuberculosis that is accurate, cheap, simple to administer and widely available is much needed. Emerging research on appropriate biomarkers to differentiate active and latent tuberculosis shows promise but remains hampered specimen collection and storage and the development of sustainable paediatric biorepositories [10]. A large systematic review has highlighted the significant heterogeneity of studies and the need for specific validation in children [11]. The availability of newer generations of IGRA such as TBGold-plus, Polymerase-chain-reaction tests and biomarkers is limited outside of specialist centres, limiting their utility.

There have been several systematic reviews of note assessing the utility of IGRA in children which concluded that TST and IGRA have similar sensitivity with differing limitations and specificity [12–15]. These studies are over 10 years-old and the data limited thereby preventing sub-analysis of children under 5 years of age [12–15]. Furthermore, one published meta-analysis of IGRA in children under 5 years of age was hindered by the limited number of studies at the time [16]. Since then, several new studies have been published, thus a review of this topic is timely.

This study aimed to establish the concordance between the widely available QFT-GIT and TST by way of pooled aggregate data, and thus to establish the utility of using QFT-GIT for diagnosing tuberculosis infection in children under the age of 5.

## Materials and methods

This systematic review and meta-analysis were conducted in accordance with the PRISMA guidelines and was registered to PROSPERO (CRD42021246577). Ethics exemption was granted under a low risk ethics pathway by the Royal Children’s Hospital Victoria HREC committee. This review included children presenting with both signs of active TB disease and those suspected of latent infection. Studies were identified by two methods: systematic database search and review of references. Systematic searches were conducted on MedLINE, EMBASE and Cochrane. Searches were restricted from 1998 till the 27^th^ June 2023 (the date when the final search from each database was conducted). The Boolean search strategy (syntax adjusted according to database) was used as displayed in Table 1.

**Table 1:** Search strategy & inclusion-exclusion criteria.

Boolean Search Strategy:
((Interferon-gamma release tests/ OR Interferon-gamma/bl [Blood] OR Tuberculin Test/ OR ((interferon* adj2 (test*1 or assay)) OR IGRA OR quantiferon*)) AND (tuberculosis/ OR latent tuberculosis/ OR tuberculosis) AND (newborn* OR baby OR babies OR neonat* OR infan* OR toddler* OR pre-schooler* OR preschooler* OR kindergarten OR boy OR boys OR girl OR girls OR child OR children OR childhood OR pediatric* OR paediatric*) AND ("Sensitivity and Specificity"/ OR "Predictive Value of Tests"/ OR "reproducibility of results"/ OR discordant))
**Inclusion Criteria**
a) The patients included in the study must be children 5 years or younger, or data for this age group must be extractable without sex restriction
b) Records must report original data
c) The reference standard must be TST
d) The index test must be QFT-GIT
**Exclusion Criteria**
a) Reviews, metanalyses, non-human research, case reports
b) Inability to extract core data points required to construct 2x2 tables for calculation of sensitivity and specificity
c) Studies that included patients co-infected with HIV or other immune compromising conditions such as malnutrition, unless data can be extracted for patients that are not immunocompromised
d) Studies that included patients treated with anti-tubercular treatment, unless data can be extracted for patients that were not
e) Studies where data in unable to be extracted due to language other than English

Identified records were downloaded to EndNote X9. The reference lists of relevant studies were searched manually to identify any additional studies that may have been eligible for inclusion. Following exclusion of duplicate studies, two reviewers (VM and TV) independently screened the titles of all identified studies for initial inclusion before screening the abstracts and full texts for inclusion in the review. Disagreements between the two primary reviewers were resolved through the involvement of a third independent reviewer. Corresponding authors of studies that were eligible for inclusion but where data or information were missing from the published manuscript were contacted. The pre-defined inclusion and exclusion criteria are described in Table 1.

The following data points were extracted and/or calculated from included studies: year of publication, country of study, sample size, age of included patients, cut off reference of TST (mm) and indeterminate rate of QFT-GIT. The True Positive, True Negative, False Positive, False Negative, diagnostic odds ratio (DOR), and sensitivity and specificity were calculated using 2 x2 contingency tables from the included studies or additional unpublished data obtained from the authors. Cohen’s kappa statistic was either extracted from the study were calculated or calculated using statistical software.

The included studies were assessed for risk of bias and quality using the Quality Assessment of Diagnostic Accuracy Studies (QUADAS) tool [17]. Data analysis was performed on SPSS, version 27 (IBM, USA) and Review Manager version 5.4 (Cochrane), and graphs were generated on GraphPad Prism version 9.0 (GraphPad software).

The ability of QFT-GIT to distinguish between disease and no disease was delineated using a Summary Receiver Operating Characteristic (SROC) curve. The area under the curve was calculated and the following classification was used to determine the utility of the test: 0.5–0.6 (unsatisfactory), 0.6–0.7 (satisfactory), 0.7–0.8 (good), 0.8–0.9 (very good) and 0.9–1.0 (excellent). In order to graphically depict the variability of results and demonstrate the weighted means of data, Forest plots were constructed. Heterogeneity between studies was assessed using Chi-square, Cochran-Q statistic and *I*^*2*^ statistic.

## Results

### Search results

**Fig 1:**
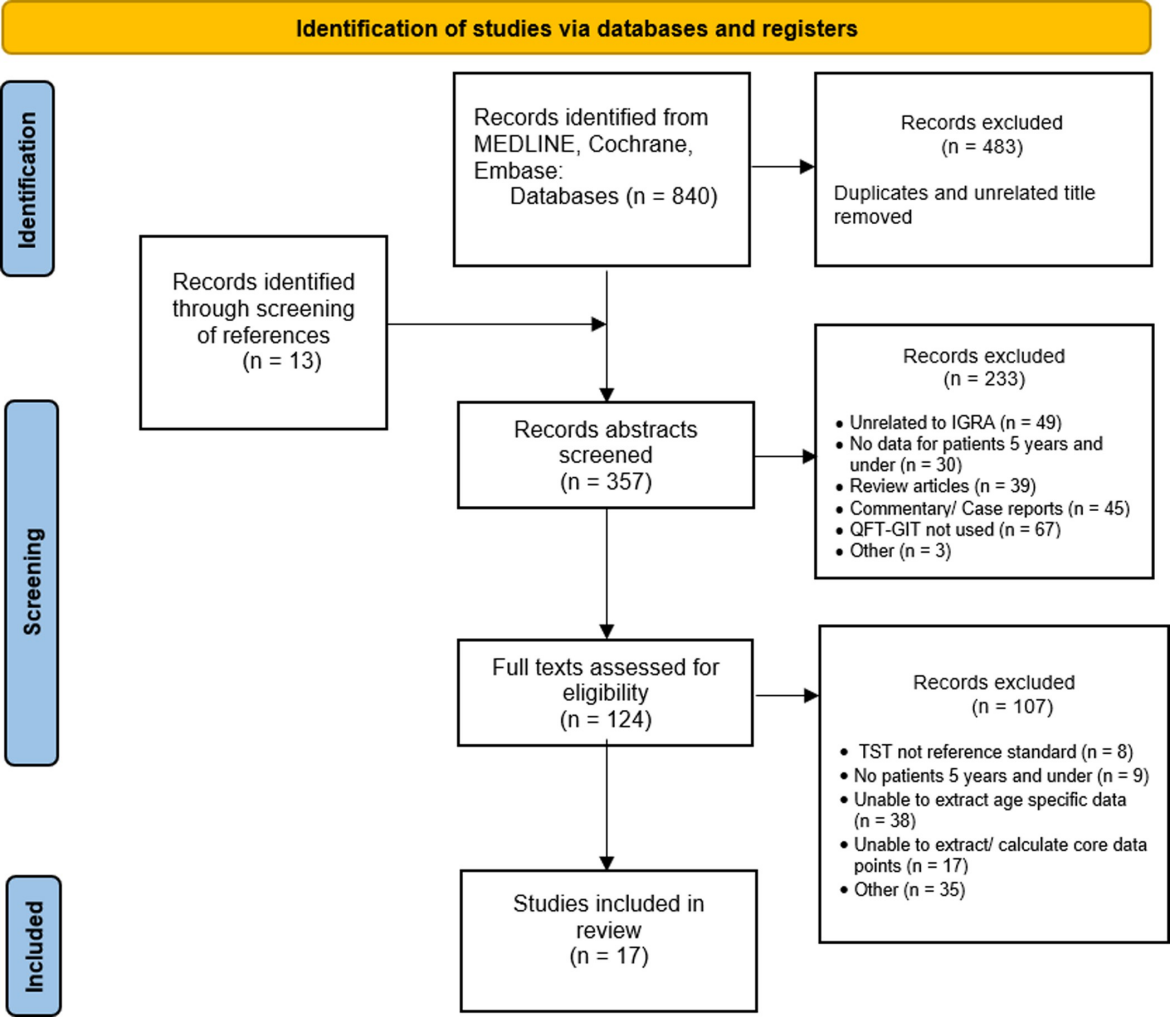
PRISMA flowchart of included studies.

The utilised search strategy revealed a total of 840 studies, of which 357 abstracts were reviewed (Fig 1). Following the exclusion of duplicates and screening of titles and abstracts, 124 studies were identified for full text review. 17 of these records were eligible for inclusion in quantitative analysis. Common reasons for discarding studies included: inability to extract data for children under 5 years of age, inability to distinguish between TSPOT-TB and IGRA test utilisation, children living with HIV, other immunocompromised states and/or malnutrition being included in studies.

### Characteristics of included studies

The characteristic of included studies [18–34] is summarised in Table 2 below. The total number of children included for this metanalysis was 4,335. The lowest number of children in a single study was 23 children. The highest number of children being included in a single study was 1,020.

**Table 2:** Study characteristics and quality assessment.

Study	Country	No. of children	QUADAS	Age Range	QFT-GIT Cut off	TST Cut off	QFT-GIT Indeterminant Rate	Proportion of sample received BCG vaccine (n/N)
Ahmed et al (2020) [18]	USA	898	12/14	0–15 years	>0.35 IU/ml	Variable	-	596/898
Bielecka et al (2017) [19]	Poland	36	14/14	0–18 years	>0.35 IU/ml	≥ 10mm	-	-
Blandinieres et al (2013) [20]	France	23	11/14	0–15 years	>0.35 IU/ml	>/ = 10mm	1/23	-
Chiappini et al (2019) [21]	Italy	253	12/14	0–5 years	>0.35 IU/ml	Variable	-	-
Debulpaep et al (2019) [22]	Belgium	59	12/14	0–5 years	>0.35 IU/ml	≥ 5mm	2/59	49/59
El azbaoui et al (2016) [23]	Morocco	33	11/14	0–15 years	>0.35 IU/ml	> 10mm	-	-
Elliot et al (2018) [24]	Australia	52	10/14	0–14 years	>0.35 IU/ml	> 10mm	-	43/68
Garazzino et al (2014) [25]	Italy	616	12/14	0–2 years	>0.35 IU/ml	Variable	-	170/616
Mastrolia et al (2018) [26]	Italy	1020	11/14	0–18 years	>0.35 IU/ml	Unclear	-	-
Moyo et al (2011) [27]	South Africa	376	11/14	0–3 years	>0.35 IU/ml	>/ = 10mm	-	100%
Okada et al (2008) [28]	Cambodia	195	12/14	0–5 years	>0.35 IU/ml	Unclear	-	-
Onur et al (2012) [29]	Turkey	23	11/14	0–14 years	>0.35 IU/ml	Variable	-	-
Pavic et al (2015) [30]	Croatia	171	11/14	0–5 years	>0.35 IU/ml	Variable	2/171	100%
Pavic et al (2011) [31]	Croatia	141	11/14	0–5 years	>0.35 IU/ml	> 10mm	1/141	100%
Rose et al (2015) [32]	Canada	45	14/14	0–18 years	>0.35 IU/ml	Variable	2/45	-
Velasco-Arnaiz et al (2018) [33]	Spain	369	12/14	0–5 years	>0.35 IU/ml	Variable	14/383	98/369
Zubarioglu et al (2019) [34]	Turkey	25	10/14	0–16 years	>0.35 IU/ml	Variable	-	24/25

The majority of studies reported an age range of 0–5 years old, with three studies having a smaller age range of 0–2 years, 0–3 years or 0–4 years. All studies utilised TST as the reference standard, although the cut-off values used for TST positivity varied across studies. QFT-GIT was used as the index test in all studies and a cut off value of >0.35 IU/ml was considered positive. All studies were conducted in middle to high income countries. They were conducted across 14 countries and 4 studies in countries with high Tuberculosis incidence (smear-positive TB >25 per 100 000 population). These diagnostic test parameters are summarised in Table 3.

**Table 3:** Study diagnostic test parameters.

Study	TP (n)	FP (n)	FN (n)	TN (n)	Sensitivity (95% CI)	Specificity (95% CI)	DOR (95% CI)	Κ-coefficient (95% CI)
Ahmed et al (2020) [18]	32	7	198	661	0.14 (0.09–0.19)	0.99 (0.97–1.00)		0.177 (0.117–0.236)
Bielecka et al (2017) [19]	5	1	1	29	0.83 (0.36–1.00)	0.97 (0.83–1.00)	145.00 (7.74–2,714.96)	0.800 (0.533–1.000)
Blandinieres et al (2013) [20]	14	0	6	3	0.70 (0.46–0.88)	1.00 (0.29–1.00)	15.62 (0.70–348.11)	0.378 (0.043–0.713)
Chiappini et al (2019) [21]	82	17	150	4	0.35 (0.29–0.42)	0.19 (0.05–0.42)	0.13 (0.04–0.39)	
Debulpaep et al (2019) [22]	5	4	2	48	0.71 (0.29–0.96)	0.92 (0.81–0.98)	30.00 (4.35–206.93)	0.567 (0.259–0.875)
El azbaoui et al (2016) [23]	8	8	6	11	0.57 (0.29–0.82)	0.58 (0.33–0.80)	1.83 (0.45–7.41)	0.148 (0.259–0.875)
Elliot et al (2018) [24]	3	5	8	36	0.27 (0.06–0.61)	0.88 (0.74–0.96)	2.70 (0.53–13.69)	0.167 (-0.140–0.475)
Garazzino et al (2014) [25]	96	25	29	466	0.77 (0.68–0.84)	0.95 (0.93–0.97)	61.70 (34.61–110.02)	0.726 (0.657–0.795)
Mastrolia et al (2018) [26]	53	9	84	874	0.39 (0.30–0.47)	0.99 (0.98–1.00)	61.27 (29.20–128.58)	0.490 (0.403–0.576)
Moyo et al (2011) [27]	57	11	13	295	0.81 (0.70–0.90)	0.96 (0.94–0.98)	117.59 (50.18–275.54)	0.787 (0.705–0.869)
Okada et al (2008) [28]	28	5	19	143	0.60 (0.44–0.74)	0.97 (0.92–0.99)	42.15 (14.53–122.28)	0.626 (0.491–0.760)
Onur et al (2012) [29]	3	3	4	13	0.43 (0.10–0.82)	0.81 (0.54–0.96)	3.25 (0.46–22.93)	0.251 (-0.173–0.675)
Pavic et al (2015) [30]	18	8	13	132	0.58 (0.39–0.75)	0.94 (0.89–0.98)	22.85 (8.33–62.67)	0.559 (0.391–0.726)
Pavic et al (2011) [31]	14	4	11	112	0.56 (0.35–0.76)	0.97 (0.91–0.99)	35.64 (9.99–127.17)	0.590 (0.405–0.775)
Rose et al (2015) [32]	8	1	3	33	0.73 (0.39–0.94)	0.97 (0.85–1.00)	88.00 (8.05–961.69)	0.744 (0.508–0.979)
Velasco-Arnaiz et al (2018) [33]	56	0	62	251	0.47 (0.38–0.57)	1.00 (0.99–1.00)	454.71 (27.7–7,461.16)	0.551 (0.460–0.642)
Zubarioglu et al (2019) [34]	11	6	1	7	0.92 (0.62–1.00)	0.54 (0.25–0.81)	12.83 (1.26–130.51)	0.448 (0.128–0.767)

TP (True Positive), FP (False Positive), FN (False Negative), TN (True Negative)

### Quality of included studies

All included studies fulfilled at least 10 of the 14 criteria listed in the QUADAS tool [17] indicating they were ‘high’ quality studies. There were however several shared shortcomings in study designs. These included: a lack of commentary on the time interval between the reference standard and index test and the blinding of test interpretation. Furthermore, bias may have been introduced into studies through sampling methods used. In some studies, TST positive cases were preferentially recruited to the study, raising concerns regarding whether the sample population is reflective of the general population.

### Meta-analysis, diagnostic accuracy of QFT-GIT

**Fig 2:**
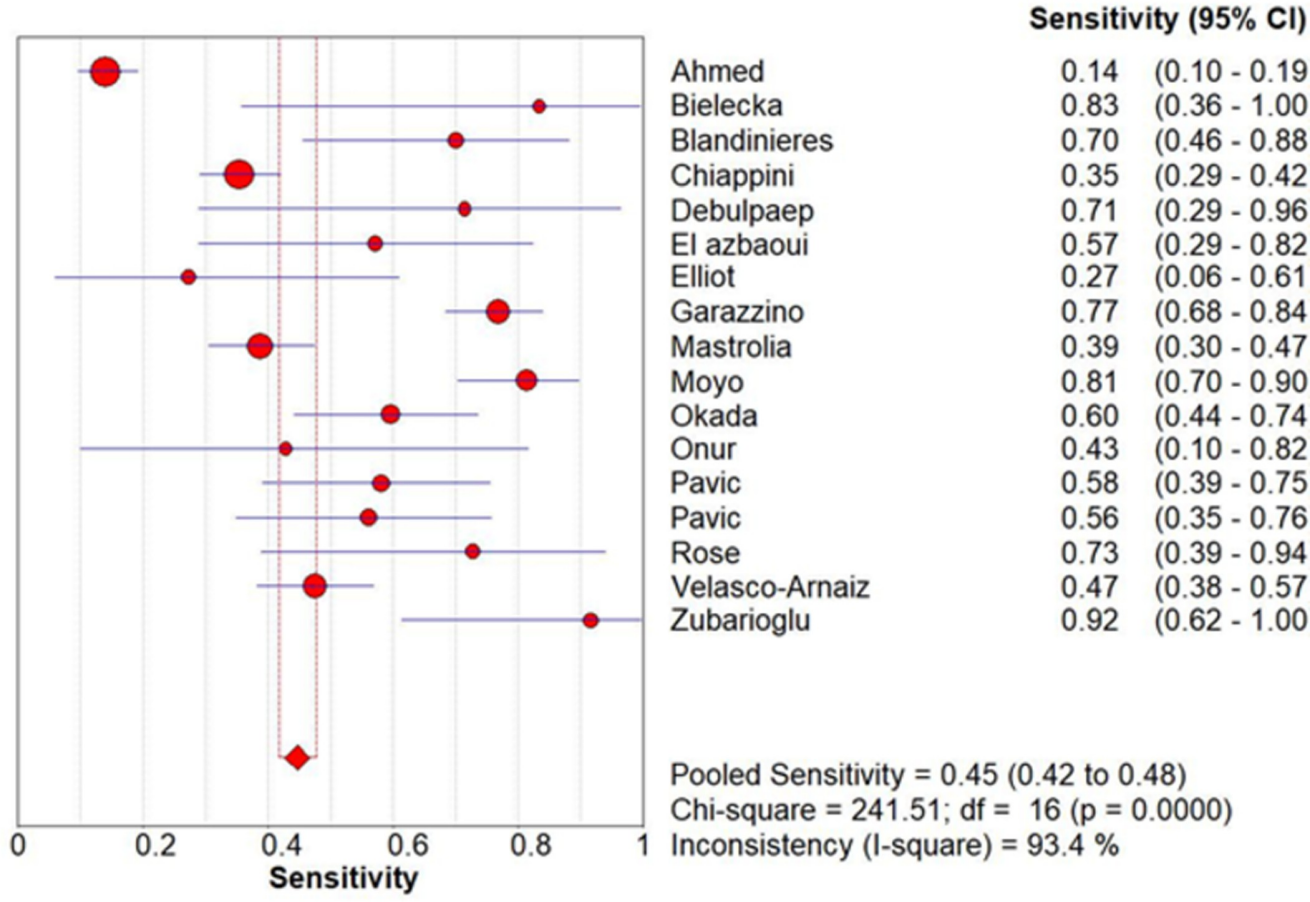
Forest plot of sensitivity.

**Fig 3:**
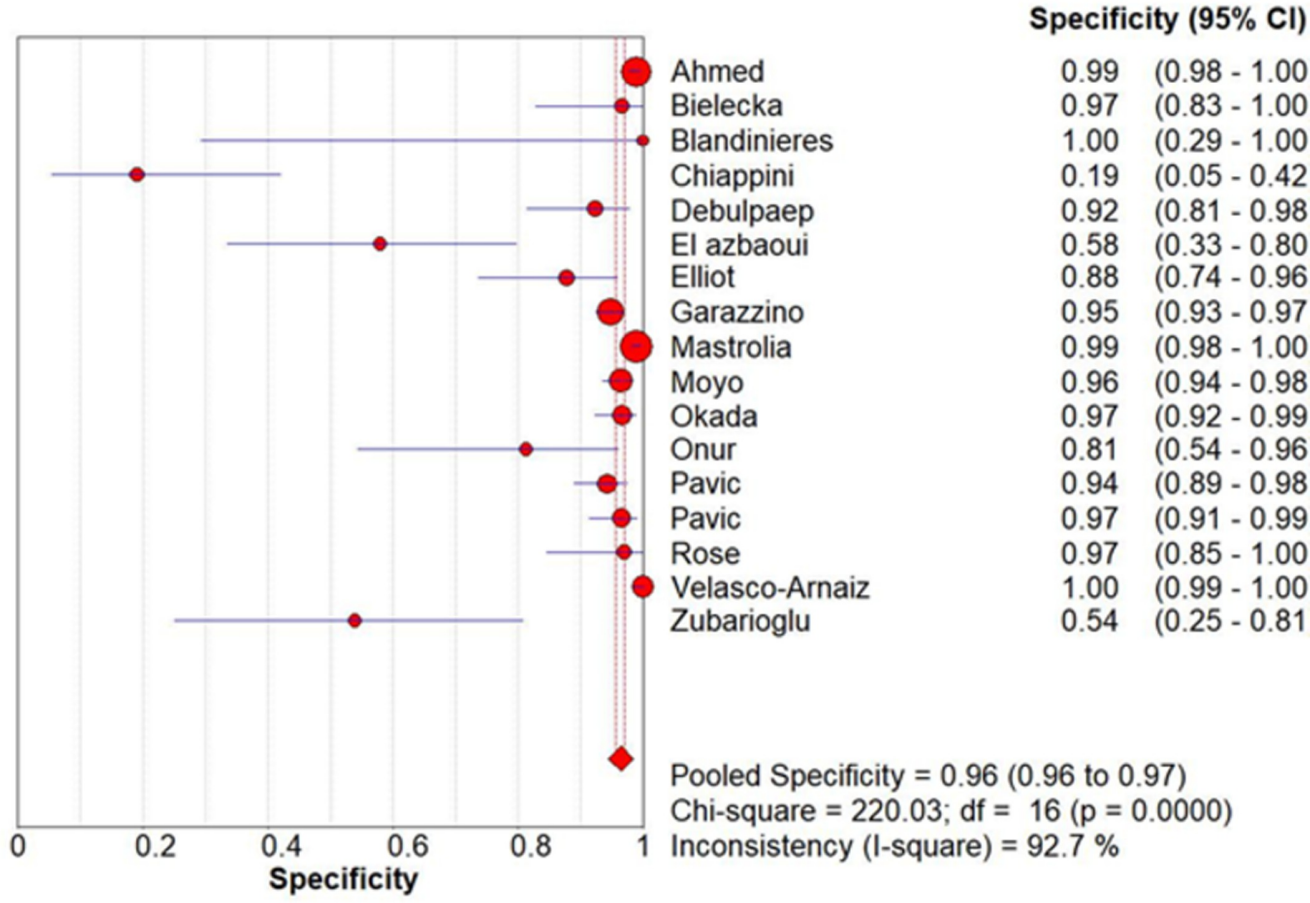
Forest plot of specificity.

All studies allowed for the extraction or calculation of sensitivity, specificity, and diagnostic odds ratio (DOR) (Table 3). The pooled sensitivity, specificity and DOR were 0.45 (0.42–0.48), 0.96 (0.96–0.97) and 18.84 (7.33–48.41) respectively, which are graphically represented below in Forest plots of sensitivity (Fig 2) and specificity (Fig 3).

**Fig 4:**
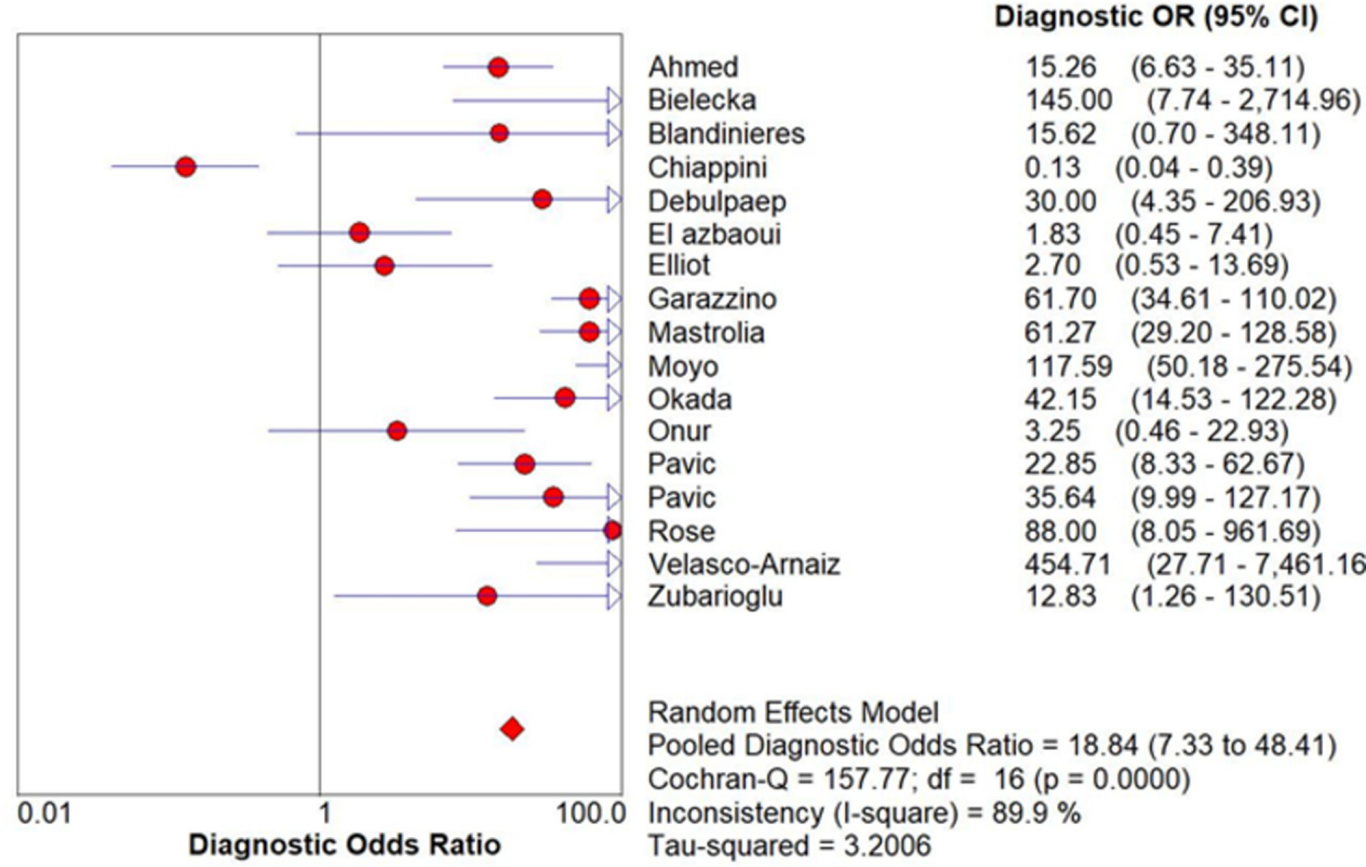
Forest plot of diagnostic odds ratio (DOR).

**Fig 5:**
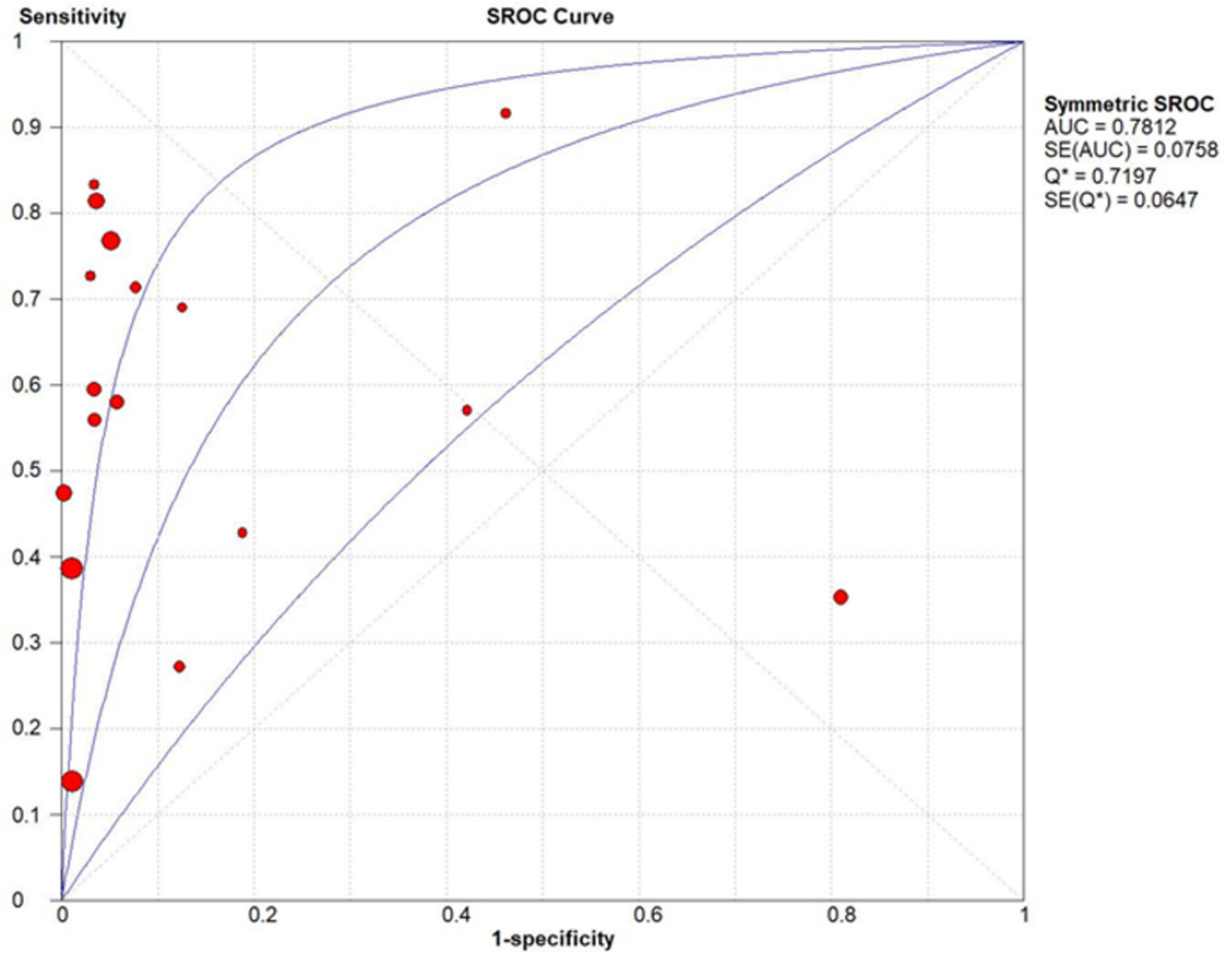
SROC curve.

Chi-square and *I*^*2*^ analysis demonstrated considerable heterogeneity between studies: sensitivity (Chi-square = 241.51, *I*^*2*^ = 93.4%), specificity (Chi-square = 220.03, *I*^*2*^ = 92.7%) and DOR (Cochran-Q = 157.77, *I*^*2*^ = 89.9%) shown Fig 4. A summary receiver operating characteristic (SROC) curve is shown in Fig 5 with each point representing a single study and the size of each point representing the relative sample size of each study. The ability of QFT-GIT to discriminate with disease and no disease was “good” as demonstrated by the SROC (Fig 5) and AUC of 0.7812 and Q* value of 0.7197.

### Meta-analysis, agreement between TST and QFT-GIT

The Kappa (k) co-efficient was calculated for each study to determine the degree of congruence between TST and QFT-GIT results. There was a large range of k co-efficient values ranging from 0.167 to 0.800, with the average being 0.501.

## Discussion

This meta-analysis has demonstrated that QFT-GIT demonstrated a “good” ability to distinguish between cases of “disease” and “no disease”, as indicated by the AUC of the SROC. However, the low pooled kappa co-efficient indicates a low degree of agreement between TST and QFT-GIT. These results contrast with a North American study whereby IGRA sensitivity was similar to TST in children <5 years of age and test sensitivity was only reduced in <2 years old, with indeterminate results higher in <1 years old [35]. The data available in the included studies QFT-GIT had a pooled indeterminant rate of 2.68%. The possibility of lowering the cut off value for QFT-GIT in this age group to increase sensitivity has garnered attention and may be a future means for improving TB diagnosis.

In the present study, only 44.6% of TST positive cases returned a positive QFT-GIT. This may represent the lower sensitivity of QFT-GIT in the 5 years and under population (i.e., a high false negative rate), or the low specificity of TST (i.e., a high false positive rate), or a combination of both. One approach to interpreting these data suggests that up to 55.4% of children could potentially miss out on anti-tuberculosis therapy if QFT-GIT was used as the diagnostic indicator. On the contrary, it may also indicate that many children receive unnecessary anti-tuberculosis therapy if TST is used as the diagnostic indicator. A more effective diagnostic test could better guide treatment choices to effectively manage morbidity and mortality, but also to ensure the optimal duration of TB infection regimens to minimise adverse effects.

The results of included studies must be viewed in the context of where they were undertaken. In high-income countries such as Australia and the USA there are two main clinical scenarios whereby children are tested for TB. They either undergo screening due to their migrant, refugee or contact status or because they exhibit symptoms compatible with Tuberculosis. The current WHO guidelines [36] suggest a negative symptoms screen as a sufficient method to rule out active TB. A thorough history and examination is insufficient when assessing for TB infection, which is not associated with signs or symptoms and often a contact history is not elicited. In such cases clinicians must have a standardised, sensitive, and specific method for identifying children with TB infection, so that they can be appropriately placed on preventative therapy.

International guidelines differ in recommendations between using TST and IGRA. American guidelines suggest performing TST over IGRA in healthy children under the age of 5 for evaluation of LTBI [37]. The authors of a major European guideline (European Centre for Disease Prevention and Control, 2011) [38] share a similar sentiment to the suggestions in American guidelines that “the simultaneous use of TST and IGRAs could be beneficial in identifying LTBI”. They also concluded that the low sensitivity of IGRAs does not support the role of IGRAs in ruling out tuberculosis infection [37]. The UK NICE guidelines [39] state that TST should be used as the preferred first-line test for investigating children for TB infection, with interferon-gamma release assays only used alone in children and young people if Mantoux testing is not available or impractical, however IGRA testing should be undertaken in children who are TST negative to improve sensitivity further. Current Australian guidelines [40] state that IGRA and TST should only be used as auxiliary tests in conjunction with radiological (e.g., chest x-ray) and microbiological (e.g., sputum samples) investigations when diagnosing active tuberculosis. Australian guidelines also state that negative IGRA/TST results are not acceptable means of excluding tuberculosis infection, although a positive result may be used to diagnose latent infection [40].

There are several potential explanations for the findings of this study. The biological basis for the reduced sensitivity of IGRAs in young children is characterised by incomplete immune maturation [41]. This is reinforced by the hugely varying sensitivities documented in previous studies, which is attributable to varying prevalence of co-morbid immunocompromising conditions such as HIV co-infection and malnutrition between studies. The immunological basis of IGRAs relies on an adequate release of interferon-gamma by T-helper 1 cells in response to antigenic stimulation which is associated with protection against *M*. *tuberculosis*, although not the sole protector in young children [42]. A potential reason for differing performance in young children is incomplete immune maturation and limited interferon gamma production [41]. It is known that infants have a T-helper 2 cell skewing of their immune system, perhaps the reason for their increased susceptibility to TB and the reduced sensitivity of IGRA [43]. The skin reaction induced by TST on the other hand, utilises both T-helper 1 and T-helper 2 immune responses, perhaps the reason for its increased sensitivity [42]. It is also for these reasons that children with co-morbid immunocompromising conditions were not included in our study. In such situations, TST and IGRA are both known to have reduced sensitivity due to the depletion of T cell responses. Furthermore, the risk of developing active and severe TB infections is much higher in these children, supporting the administration of anti-tuberculosis therapy based on clinical suspicion alone (risk of severe TB infection outweighs the risk of anti-tuberculosis drug-associated adverse effects). As such, the choice of administering treatment may not be so heavily dependent on TST/IGRA testing as in the case of immuno-competent children.

There are several limitations in interpreting this study. An important potential imitation in these studies is that defining true TB infection in the absence of a gold standard is challenging. This was highlighted by the different definitions for TB infection and LTBI adopted across studies. We considered TST positivity to be an indicator of true TB infection, as it remains the preferred diagnostic tool in many jurisdictions [36–38, 40] and to focus on TB infections as a whole spectrum condition due to the implications of treatment. It is important to consider the limitations of using TST as the gold standard. Indirect data demonstrating low specificity in BCG vaccinated children and the potential for reporting IGRA sensitivity inaccurately low should be considered in each individual clinical context. As detailed in Table 2, BCG status was not always reported and varied significantly when reported across these studies. All 5 studies [22, 27, 30, 31, 34] with high BCG rates (>75%) concluded that TST and QFT-GIT should be used cautiously in conjunction and positivity of either test suggests infection.

In addition, varying definition of cut-off values complicates the comparison of data between studies and may account for some of the high heterogeneity between studies. This variability limits discussion regarding individual study conclusions. In the two studies that included only active tuberculosis, one concluded that QFT-GIT was more sensitive [23] and the other suggested both tests be used as neither had an individual sensitivity high enough to exclude tuberculosis [34]. We accepted varying TST cut-off values, as individual jurisdictions ascertain local cut-off values based on a range of factors that can affect TST reactions [44]. The high degree of heterogeneity between study designs (especially inclusion and recruitment) made sub-analysis of BCG vaccinated cases or those with differing TST cutoffs unfeasible. Further directed research designed to undertake these sub-analyses would be of high value.

Indirect measures of IGRA test performance such as correlation with exposure gradient have been observed in children, however sensitivity in active disease against a microbiologic standard established in adults has been questioned in children [45]. QuantiFERON-TB Gold testing has been shown in different context to identify false positive TSTs, such as low risk school age children [44]. Longitudinal studies exploring progression rates from untreated children with discordant screening results could provide more robust indirect data of test performance in this group. However, a recent systematic review confirms these studies are sparse in children and often from heterogenous population groups, however pooled data did not show evidence indicating QFT-GIT was better or worse than TST at a cut off of 5mm [46].

Generalisability of results across different income settings is another potential limitation of this study, as most studies were performed in middle and high-income countries. QFT-GIT is likely to be inaccessible (due to need for laboratory services) in low-income settings where the clinical utility of the test may be impractical. A key consideration in the utility of these tests is the indeterminate rate of QFT-GIT and the inter-clinician variability in the reading of TST skin reactions [47]. Although this comparison is beyond the scope of this review, from the data available in the included studies QFT-GIT had a pooled indeterminant rate of 2.68%. This is comparable to the quoted indeterminate rate of less than 2% in older age groups.

Given the widespread availability of QFT-GIT in the Australian context, we focused on diagnosis of tuberculosis infections in children aged 5 years and under using this test. The utility of the alternative commercially available test the T-SPOT.TB test (Oxford Immunotec, Oxford, UK), which does not utilise the additional antigen of TB7.7, was beyond the scope of this review. Whilst a newer version, QFT plus (which incorporates a fourth antigen), is available, there are insufficient data published to conduct a meta-analysis in the 5 years and under population. Early studies shown limited (moderate) concordance with both QFT plus and TST recommended in under 5-year-olds in one study [48].

## Conclusion

Children aged 5 years and under pose a diagnostic dilemma when it comes to tuberculosis and the use of QFT-GIT. This is partly driven by the underdeveloped immune system of young children, and partly due to the lack of a gold standard to allow for the accurate assessment of new diagnostic tools being developed. Our findings support the general advice promoted by several guidelines, in that QFT-GIT should be used and interpreted in a judicious manner in the 5 years and under age group. Results should be interpreted with caution due to uncertainty, risk of bias and unexplained heterogeneity across studies. Due to the lower sensitivity of QFT-GIT in comparison to TST, a negative result should not be considered as an absence of TB infection. A positive result however, could allow for the delineation of TST positive children who may benefit the most from anti-tuberculous therapy due to improved specificity. Nonetheless, from the current evidence, QFT-GIT is a potentially helpful additional test, particularly in BCG vaccinated children but should not be used as a sole test to exclude tuberculosis in this age group. Diligent clinical acumen is required when making decisions regarding management based on these tests.

## Supporting information

S1 Checklist PRISMA 2020 checklist. (DOCX)
